# Free to help? An experiment on free will belief and altruism

**DOI:** 10.1371/journal.pone.0173193

**Published:** 2017-03-10

**Authors:** Job Harms, Kellie Liket, John Protzko, Vera Schölmerich

**Affiliations:** 1 Department of Economics, Erasmus University, Rotterdam, the Netherlands; 2 Department of Psychology and Brain Sciences, University of California Santa Barbara, Santa Barbara, California, United States of America; 3 Department of Social and Behavioural Sciences, Erasmus University College, Erasmus University, Rotterdam, the Netherlands; Middlesex University, UNITED KINGDOM

## Abstract

How does belief in free will affect altruistic behavior? In an online experiment we undermine subjects’ belief in free will through a priming task. Subjects subsequently conduct a series of binary dictator games in which they can distribute money between themselves and a charity that supports low-income people in developing countries. In each decision task, subjects choose between two different distributions, one of which is more generous towards the charity. In contrast to previous experiments that report a negative effect of undermining free will on honest behavior and self-reported willingness to help, we find an insignificant average treatment effect. However, we do find that our treatment reduces charitable giving among non-religious subjects, but not among religious subjects. This could be explained by our finding that religious subjects associate more strongly with social norms that prescribe helping the poor, and might therefore be less sensitive to the effect of reduced belief in free will. Taken together, these findings indicate that the effects of free will belief on prosocial behavior are more nuanced than previously suggested.

## Introduction

“We must believe in free will, we have no choice.”

- Isaac Bashevis Singer–

### Free will belief and social behaviors

Do humans have free will? This is the topic of an ancient debate that remains unresolved up to this day [1–4]. The implications of this debate extend beyond the intellectual realm. Despite the lack of consensus in the academic community, people young and old across the world believe that they have free will [5,6] and most people even believe they have more free will than others [7]. Some scholars argue that widespread belief in free will has evolved as it allows for larger and more complex societies to function and thrive [8]. Instilling in people a sense of control over their actions, this belief has allowed for the justification of rules and institutions that punish anti-social behavior and reward pro-social behaviour. Thus, rather counter-intuitively, the belief in free will is proposed to have enabled humans to become better at adhering to social norms.

Greater belief in free will has been associated in observational research with a range of positive outcomes, including better career prospects and higher job performance [9]. Furthermore, experimental evidence also points towards benefits of greater belief in free will, for example by promoting appreciation towards acts of kindness by others, who were “free” to also be unkind [10].

However, the level of people’s belief in free will and their locus of control [i.e. the extent to which people attribute control to themselves vs. their environment] have been declining in recent decades [11]. Belief in free will and locus of control are strongly correlated [9] and conceptually related: without a belief in free will it is more difficult to attribute control to oneself. The decline of these beliefs coincides with the popularization of insights from neuroscience, for example the famous Libet experiments [12] which conclude that free will is an illusion [13,14]. Neuroscience experiments by Libet and others–the so-called ‘willusionists’ [15], demonstrate that information about brain activity can be used to predict decisions before the decision-maker becomes aware of making a decision. Various philosophers contest the claim that free will does not exist [4,16]. Regardless of whether the inferences from neuroscientific evidence to the supposed impossibility of the existence free will are correct, the changing attitudes in society about free will and self-control have been shown to influence various social behaviours.

Various lab experiments have undermined people’s belief in free will by priming tasks, in which subjects read texts about neuroscientific evidence implying the non-existence of free will. These studies tend to show that exposure to such primes undermines honesty and willingness to help others. One study shows that undermining belief in free will causes increased cheating in tasks where subjects could earn more money by lying [17]. A study by Baumeister and colleagues 2009 finds that experimental reduction of belief in free will through a reading task lowers people’s likelihood of reporting to be willing to help others in various hypothetical scenarios [18]. It should be noted that this study did not look into the relationship between free will disbelief manipulations and *actual* helping of others. This question has yet to be empirically investigated.

Next to their experimental findings, Baumeister and colleagues 2009) also reported that subjects with a stronger *dis*belief in free will were less likely to sign up for volunteer work absent of experimental manipulations). One interpretation for these findings is that a disbelief in free will gives people an excuse to justify their selfish tendencies [19]. In other words, people can refrain from engaging in prosocial behavior and then justify this by explaining that they have very little control over their own behavior. Further supporting these findings, studies show that undermining free will belief reduces people’s ability to control themselves [20–22]. This reduced self-control would, in turn, diminish people’s willingness to act prosocially [18]. Taken together, the current evidence to support the view that free will disbelief undermines prosocial behavior is still rather limited.

While studies have shown that undermining free will belief leads to increased cheating and reduced likelihood of reported willingness to help others, other studies have found that undermining free will belief can actually promote sympathy for others. For example, lowered free will beliefs have been shown to reduce the attribution of blame of criminal offenders [23,24]. This finding suggests that undermining people’s belief in free will increases their perception that other people are shaped by forces outside of their own control, such as their genetic composition or their upbringing. Indeed, another experiment shows that the tendency to blame others depends on the perceived level of control that others have. In this experiment [25], subjects were shown a video in which an ‘active’ person interacted with another ‘passive’ person, from whom they could steal money. In both treatments the active person stole the entire endowment [$10] from the passive person. In one treatment, this action was the result of a random process outside of the control of the active person [die roll], whereas in the other treatment it was the result of an active choice made by the passive person. The observing subject was then asked to judge the blameworthiness of the active person, and the authors [ibid] found that more blame was attributed in the condition were an active choice was made. In a similar vein, Fong [26] finds that people, even those that perceive themselves as being unconditionally altruistic, donate more to welfare recipients if they are informed that these recipients are actively looking for work as opposed to waiting for a work opportunity to arise. The results of these suggest that undermining free will belief may also promote prosocial behavior by increasing people’s perception of the lack of control other people have on their own lives and consequently increasing their willingness to help.

Another open question is whether the effects of free will beliefs on social behavior are homogenous across different groups. Various studies have shown that a range of behavioral patterns observed in lab experiments with college students do not generalize to other populations that are less educated, rich and westernized [27,28]. Since all previous experiments with free will manipulations were conducted among college students, it remains unclear how universal the effects of free will beliefs on social behavior are. One group that might respond differently to free will manipulations are religious people, as belief in free will is higher among religious people. Various studies have shown that religious affiliation is associated with higher charitable giving [29], higher propensity to volunteer to help the poor and elderly [30] and higher giving to charities in dictator games–an activity where subjects are given a sum of money and can decide how much they want to donate to another subject [15]. It should be noted that these studies apply to western countries where Christianity is the main religion. Furthermore, experimental studies provide causal evidence to support the theory that religious primes can promote honesty and prosocial behavior, both in the lab [31,32] and in the field [33]. Moreover, there is some evidence that religious primes have different effects on social behaviors according to the religious status of subjects. For example, the willingness to engage in costly punishment of free-riders in a public-goods game was increased by a religious prime, but only among subjects that had previously made religious donations [34].

To summarize, there is currently mixed evidence on whether undermining free will belief will lead to more prosocial behaviour, it is not clear how this effect will play out amongst a diverse population, and there is no experimental evidence on whether undermining free will belief influences people’s actual likelihood of helping others. In this study we tested the effects of free will manipulations on actual behaviour among a diverse population. We did this by conducting an online experiment using a dictator game with subjects recruited via the Amazons mTurk platform.

One of the most commonly used methods to study actual [as opposed to self-reported] prosocial behavior is the dictator game. In this two-player game, one subject–the dictator—is given a sum of money, and can decide how much of this money they want to donate to the other person playing–the recipient. This recipient is passive and can do nothing but accept whatever fraction of the sum of money they are given. Under conditions of anonymity, the rational strategy for a purely selfish dictator is to give nothing, but a meta-analysis with data from hundreds of dictator game experiments shows that people donate on average between 25–30% of their money [35]. Given its simplicity, the dictator game is a useful tool to study the factors that shape prosocial behavior.

The Amazon mTurk platform allows people to earn money by completing small tasks see Methods—Procedures for more details and is increasingly used in social science experiments [36,37] and provides access to a more population that is more diverse than college students in terms of demographics, socio-economic and cultural background. For example, an experiment on mTurk used a dictator game with subjects in the U.S. and India, and found that the latter group were more sensitive to the size of the endowment [38]. Another dictator game experiment with subjects from different countries recruited through mTurk found substantial heterogeneity in dictator game play across cultures [39]. Furthermore, such experiments can be used not only to test whether behavior in standardized experiments differs across groups, but also whether these various groups respond differently to experimental manipulations.

### Research question

Our paper aims to address the following question: “How does undermining belief in free will affect altruistic behavior?” We measure altruism in terms of behavior in a binary dictator game where subjects can divide money between themselves and a charity. In line with several previous experiments about free will belief, we hypothesize that undermining belief in free will make people less inclined to engage in charitable giving. Furthermore, we hypothesize that this manipulation could have different effects among a more diverse sample of subjects.

## Methods

### Procedure

The subjects were recruited via Amazon mechanical Turk (mTurk), a crowdscourcing website that is increasingly used in the social sciences. Although providing less control over experimental conditions than lab experiments, various studies show that results obtained through mTurk are comparable to results from the lab [40–42]. At the onset of the experiment subjects were informed that the study was about the effect of exposure to text on happiness. The purpose of this was to reduce socially desirable responses due to observer bias and to prevent subjects from making a connection between the manipulation we performed and our dependent variable. Following the introduction to the experiment, subjects were asked to rate their happiness on a 1–10 scale. Subsequently, subjects were exposed to the treatment or control text. To ensure that subjects read this text, they could only click to the next page one minute after opening the page with the treatment or control text. Furthermore, subjects were requested to write a short summary of the text and they were informed that their payment could be affected if they did not do so. They were then again asked to rate their happiness. Subsequently, they continued to a set of 24 decision tasks, followed by a short survey. We explain the treatment/control text, the decision tasks and the survey in more detail below.

### Treatment—free will disbelief manipulation

The treatment consisted of subjects being asked to read a 1-page article from the popular science journal “NewScientist” in which neurological scientific evidence is presented to support the notion that humans do not have free will. The control group was shown another 1-page article from the same magazine about sustainable energy technologies (see S1 Appendix for the full text of both treatment and placebo). Subjects were asked to write a 1–2 sentence summary of the text in order to demonstrate that they had carefully read the text. This free will manipulation has successfully been used in previous studies, e.g. [23].

### Decision tasks–binary dictator games

In a second step of this experiment, subjects were told that they could allocate monetary tokens to themselves or to GiveDirectly, a charity that provides direct cash transfers to low-income households in sub-Saharan Africa. Subjects were informed that these cash transfers would be given to “people like Beatrix”, followed by a short description of this woman’s situation accompanied by a photograph of the illustrative recipient and her two children. This information was taken from the website of the charity. The exact wording to describe the example recipient was as follows: “An example of a family benefiting from GiveDirectly is Beatrice (31yrs) and her two young children, living in Kenya.” Subjects then completed 24 binary Dictator Games (DGs), which are a widely used tool to measure social preferences [43,44]. In our experiment, each binary DG consisted of two different distributions of tokens between the subject (i.e. the dictator) and the charity. For example, subjects could choose between option A) keep 50%, give 50% to charity or option B) keep 0%, give 100% to charity. Henceforth, we refer to each DG as a ‘decision task’. The number of experimental tokens that could be earned per task ranged between 0–60, with a conversion rate of 1 dollar cent/token. In other words, subjects had the chance of earning up to 60 cents per task. Previous research on mTurk has shown that dictator games with stakes of max. $1 yield similar outcomes to higher stakes [38] To ensure that subjects had an incentive to reveal their true preference in each task as they were informed that one of the games would be randomly selected at the end of the experiment and then paid out according to the choices the dictator made [45]. The order of the decision tasks was randomized to control for order effects. Subjects were informed that they had 10 seconds per task. They were also informed that if they would not choose within this timeframe, then they or the charity would not receive any money in case this task was randomly selected to be played for real money at the end of the experiment.

In each decision task, one of the two options provided a higher payoff to the charity but a lower payoff to the dictator (see Table 1). In our analysis, we classify this as the more altruistic option. The first 12 decision tasks consisted of choices between an equal distribution and an unequal distribution. For example, the fair allocation for task 1 is option A: 50% dictator/50% charity and the unfair allocation is option B: 100% dictator/0% charity. The second set of 12 tasks consisted of choices between two unequal allocations. For example, task 13 option A was 100% dictator/0% charity and option B was 0% dictator/100% charity. Decision tasks also differed in terms of whether the more altruistic option consisted of the same, less or more money than the less altruistic option. For example, in task 22, option A was 60 for the dictator vs. 20 for the charity, and option B was 0 for the dictator vs. 100 for the charity. By varying the size of the allocation we wanted to investigate whether the treatment effect was greater for options where the less altruistic option was more efficient, i.e. resulted in larger potential earning. Moreover, we varied whether or not both options provided at least some payoff to both players. In this way we wanted to investigate whether the treatment had a greater effect when dictators could “excuse” their selfish behavior by selecting an option that provided at least some payoff to the charity.

**Table 1:** Overview of decision-tasks.

Task	Option A		Option B	
	Self	Charity	Self	Charity
1	50	50	100	0
2	50	50	80	20
3	50	50	20	80
4	50	50	0	100
5	60	60	100	0
6	60	60	80	20
7	60	60	20	80
8	60	60	0	100
9	40	40	100	0
10	40	40	80	20
11	40	40	20	80
12	40	40	0	100
13	100	0	0	100
14	100	0	20	80
15	80	20	0	100
16	80	20	20	80
17	100	20	0	100
18	100	20	20	80
19	80	40	0	100
20	80	40	20	80
21	80	0	0	100
22	80	0	20	80
23	60	20	0	100
24	60	20	20	80

### Questionnaire

After the decision tasks, subjects were asked to indicate their sex, age, and perceived socio-economic status. In addition subjects were asked whether they identified with a religion: Christianity, Islam, Hinduism, Buddhism, Judaism, other, or no religion. Subjects who responded to not identify with a religion were labelled as non-religious. After presenting these survey questions we also asked subjects to indicate their self-reported level of free will, measured on a 100-point scale. This question served as a manipulation check. In addition, subjects were asked if they considered whether recipients of the charity had control over their personal situation and whether they thought that “one ought to help” people such as these recipients (see ‘Mechanisms’ in the Results section for exact phrasing). This last item allowed us to investigate whether adherence to social norms might moderate the effect of the free will disbelief treatment on altruistic behavior. For more details about these items please see S1 Appendix.

### Subjects and randomization

Subjects were recruited from U.S. members of Amazon mTurk in August 2016. As can be seen in the ‘Full sample’ column of Table 2, a total of 108 subjects participated. This sample consisted of 64% females. The average age was 34.2 years. Approximately half of our subjects considered themselves religious, the majority of religious subjects subscribing to various Christian denominations. Moreover, most subjects report to perceive their own socio-economic status to be right in the middle between most and least successful. Differences between treatment and control group in terms of age and sex were not statistically significant. However, a Mann-Whitney U-test shows that our random assignment did result in a higher fraction of non-religious subjects being allocated to the treatment group (Pr.>|z = 0.0075) as well as a higher level of self-perceived socio-economic status in the treatment group (Pr.>|z = 0.0928). To account for this imbalance we add these variables as controls in our regression analysis.

**Table 2:** Summary statistics.

	Control			Treatment			Full sample		
	Obs.	Mean	SD	Obs.	Mean	SD	Obs.	Mean	SD
Female (1 = yes)	51	0.59	0.5	57	0.68	0.47	108	0.64	0.48
Age (years)	51	33.53	11.59	57	34.81	10.3	108	34.2	10.89
Socio-econ. status (0–10 scale)	51	4.94	1.61	57	5.47	1.6	108	5.22	1.62
Non-religious (1 = yes)	51	0.63	0.49	57	0.37	0.49	108	0.49	0.5
Religious (1 = yes)	51	0.37	0.49	57	0.63	0.49	108	0.51	0.5

### Ethics statement

All participants in the experiments reported in the manuscript were informed: first, about the protocols of the study that ensure anonymity and confidentiality; second, about the content of the experiment (and the potential monetary earnings) prior to participating. Written consent was obtained from all participants included in this paper. Only those who accepted completed the experiment. Those who did not accept did not continue the experiment. Anonymity was always preserved as participants signed up through their Amazon mTurk account number. No association was ever made between their real names/addresses and the results. As is standard in socio-economic experiments, no ethic concerns are involved other than preserving the anonymity of participants. This procedure (including the consent process) was checked and approved by the Office of Research and Human Subjects at the University of Santa Barbara, the institution hosting the experiments.

## Results

### Manipulation check

For the full sample, the average level of free will belief was 69.9 on a 0–100 scale (SD = 23.6), with 100 indicating total agreement with the statement "I fully believe I have free will", and zero indicating full disagreement. The average level of free will belief in the control group was 72.2 (SD = 22.2), and in the treatment group it was 67.8 (SD = 24.8). To estimate the effect of the treatment on self-perceived free will, we run an OLS regression of the treatment on the free will measure, whilst controlling for religiosity, demographics and self-perceived socio-economic status. In line with previous studies that used similar primes to induce free will disbelief, for example (23), we find that our treatment significantly reduced self-reported belief in free will by 8.9 points on a 100 point scale, as indicated in Table 3.

**Table 3:** Manipulation check.

	Free Will Scale (0–100)
Treatment "free will disbelief"	-8.930**
	(4.115)
Female	0.278
	(4.511)
Age (30–39 years)	2.780
	(4.988)
Age (40–49 years)	3.617
	(5.822)
Age (50–59 years)	13.850
	(8.380)
Age (60+ years)	-21.565***
	(7.952)
Socio-economic status	6.808***
	(1.348)
Religious	0.256
	(4.297)
Constant	37.306***
	(9.827)
Observations	108
R-squared	0.264

*** p<0.01,

** p<0.05,

* p<0.1

OLS regression with robust standard errors clustered on subjects in parentheses. The dependent variable is the level of agreements subjects reported on a 0–100 scale to the statement "I fully belief I have free will". Age is a categorical variable with reference group = age 18–29 years. Religiosity is a dummy variable with value = 1 if subjects reported to yes to the question “Do you consider yourself as belonging to any particular religion or denomination?” The variable socio-economic status indicates on a 0–10 scale were subjects perceive themselves to be on the ladder of success in society (see S1 Appendix for more details).

### Treatment effects

**Fig 1:**
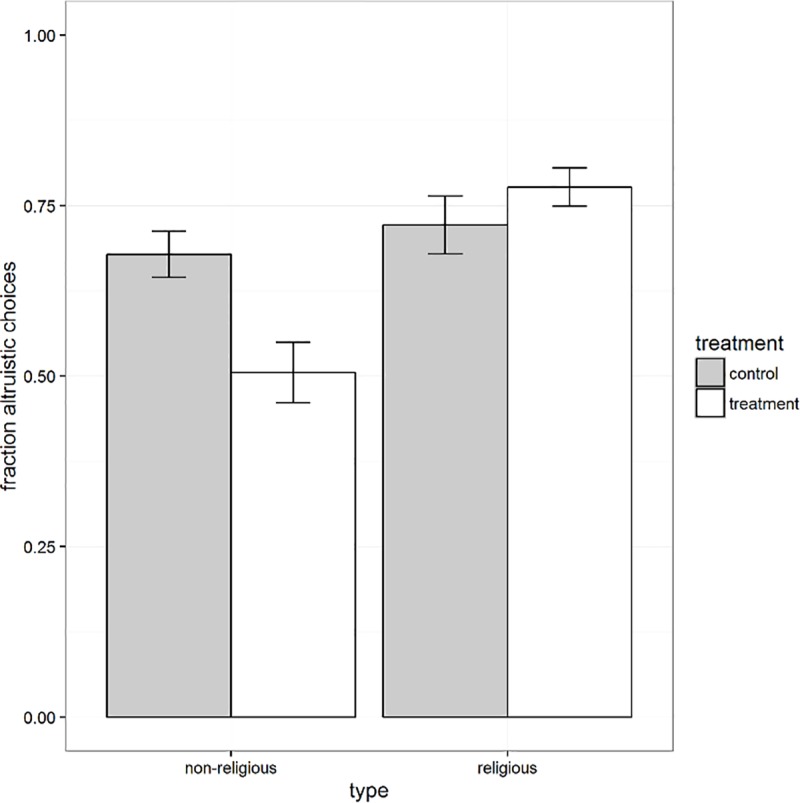
Treatment effect by religiosity. Y-axis shows fraction of altruistic choices. Error bars with 95% confidence intervals

First, we estimate the effect of the treatment on the fraction of decision tasks in which the more altruistic distribution was chosen. To this purpose, we compute for each subject this fraction. We then regress this fraction on the treatment, controlling for subject characteristics. As can be seen in columns 1–2 of Table 4, the treatment did not have a significant effect on the fraction of altruistic choices for the pooled sample. We do find that–absent the treatment—religious subjects selected a higher fraction of altruistic choices than non-religious subjects. Furthermore, when we compare treatment effects for religious and non-religious subjects, as shown in columns 3–4, we find that the treatment did result in a statistically significant fraction of altruistic choices for the non-religious subjects, of more than 21 percentage points (P<0.05), whereas there was no significant effect for non-religious subjects. This result is also reflected graphically in Fig 1.

**Table 4:** OLS regression of treatment effects.

	Fraction of altruistic choices			
	(1)	(2)	(3)	(4)
	full sample		religious	non-religious
Treatment "free will disbelief"	-0.016	-0.058	0.091	-0.214**
	(0.061)	(0.062)	(0.087)	(0.087)
Level of free will belief	0.000	-0.002	-0.001	-0.001
	(0.001)	(0.001)	(0.002)	(0.002)
Controls (age, sex, religiosity, socio-economic status)	No	Yes	Yes	Yes
Constant	0.694***	0.370***	0.609***	0.196
	(0.107)	(0.134)	(0.183)	(0.167)
				
Observations	107	107	54	53
R-squared	0.001	0.186	0.293	0.299

*** p<0.01,

** p<0.05,

* p<0.1

The dependent variable is what fraction of subjects’ choices was for the more altruistic option. Robust standard errors clustered on subjects in parentheses. The variable “Treatment free will disbelief”” has value = 1 if subjects were primed with the no-free-will story.

We then consider the subjects’ choice behavior for each individual choice task. To this purpose we use a random effects probit model, with robust standard errors clustered on individuals. The main outcome variable in these regressions is a dummy variable indicating whether or not the subject selected the more altruistic of the two options (1 = yes, 0 = no).This model allows us to test not only the treatment effect, but also control for specifics of the choices task. In particular, we control for: (i) whether the choice was between two unequal distributions or between an unequal vs. an unequal distribution, (ii) whether one of the choices was more efficient in terms of the total amount to be distributed, (iii) the cost of altruism–i.e. how much extra the subject could earn from selecting the less altruistic option, (iv) the benefit of altruism–i.e. how much extra the charity could earn if the subject selected the more altruistic option. Furthermore, we control for subject characteristics and task order In additional analyses, we also control for moral self-licensing effects as studied in a 2013 paper by Brañas Garza et al. [46] by including a lag of the variable “altruistic choice” as a control variable in a probit model without random effects. However, we find that no indication of moral self-licensing, as the coefficient on the lag variable is statistically significant and positive (results can be obtained upon request).

In line with the results from the OLS regression model we find that only among the sub-group of non-religious subjects did the treatment reduce the propensity of subjects to choose the more altruistic distribution, as can be seen by the negative coefficient on the variable “Treatment free will disbelief” in Table 5, column 4. The treatment reduces the propensity of non-religious subjects to select more altruistic option by over 25% (significant at P<0.05), whereas for religious subjects the treatment had no significant effect.

**Table 5:** Probit regression of treatment effects.

	Altruistic choice (1 = yes)			
	(1)	(2)	(3)	(4)
	full sample		religious	non-religious
Treatment "free will disbelief"	0.001	-0.096	0.070	-0.254**
	(0.084)	(0.076)	(0.080)	(0.105)
Level of free will belief	0.001	-0.002	-0.001	-0.001
	(0.002)	(0.002)	(0.002)	(0.002)
Unequal vs. unequal		0.119***	0.103***	0.128***
		(0.030)	(0.039)	(0.046)
Selfish option efficient		-0.067***	-0.055***	-0.076***
		(0.016)	(0.017)	(0.028)
Cost of altruism		-0.000	0.000	-0.001**
		(0.000)	(0.000)	(0.001)
Controls (age, sex, religiosity, socio-economic status, task-order)	No	Yes	Yes	Yes
Subjects	108	108	55	53
Observations	2,532	2,532	1,292	1,240
Wald χ^2^	0.19	77.54	50.49	36.80

*** p<0.01,

** p<0.05,

* p<0.1

Marginal effects of probit model with subject random effects. The dependent is whether or not the subject selected the more altruistic distribution (value = 1 if yes). Standard errors clustered at the subject-level. Observations where subjects did not make a decision within 10 seconds (n = 60) were excluded from this analysis. The variable “unequal vs. unequal” has value = 1 if both options consisted of unequal distributions, (e.g. 100/0 vs. 0/100) and value = 0 if only 1 option consisted of an unequal distribution (e.g. 50/50 vs. 100/0). The variable “selfish option” efficient has value = 1 if the total number of tokens to be distributed was greater in the less altruistic option, and value = 0 if the amount of tokens was equal in both options. The variable “cost of altruism” indicates the difference between the two options in terms of the number of tokens that could be earned by the dictator.

Furthermore, we find that subjects were more likely to choose the altruistic distribution in choices between two unequal distributions–one favoring the dictator and one favouring the recipient—than in choices between one equal and one unequal distribution. In addition, we find that subjects are less likely to choose the more altruistic distribution when this option is inefficient—in terms of the total size of the pie–in comparison to the other distribution. This effect occurs for both religious and non-religious subjects. Finally, we find that among non-religious subjects the decision to choose the more altruistic option is also significantly influenced by the size of its costs (to the subject) and benefits (to the recipient), as indicated by the positive and negative coefficients in column 4 on the variables “cost of altruism” and “benefit of altruism” respectively.

### Thou shalt help?

To investigate whether helping norms might moderate the effect of free will beliefs, subjects were asked to indicate which of the following two statements they most agreed with:

- *“One ought* *to help people such as Beatrix” [Beatrix is the name of example recipient of charity]*
- *“People such* *as Beatrix ought to help themselves”*.

Subjects who agree more with the former statement were labeled as “helping norm adherents”. Using a probit analysis in which we control for demographics, self-reported socio-economic status and the treatment, we find that non-religious subjects are approximately 10% less likely to adhere to the helping norm than their religious counterparts (P>|z| = 0.098), please see Table 6 below. Given the limited effect size we note that this result is merely suggesting that helping norms play a role in moderating the effect of free will disbelief among religious people.

**Table 6:** Probit analysis of determinants of helping norm.

	Adherence to helping norm (1 = yes)
Treatment "free will disbelief"	-0.083
	(0.065)
Free will scale	-0.002
	(0.001)
Religious (1 = yes)	0.101*
	(0.061)
Controls (age, sex, socio-economic status)	Yes
	
Observations	108
Pseudo-R^2^	0.1857

*** p<0.01,

** p<0.05,

* p<0.1

Note: Marginal effects of probit estimation with robust standard errors clustered on subjects in parentheses. The dependent variable is a dummy indicating whether the subject identified more with the statement “one ought to help poor people” (= 1) or with the statement “poor people ought to help themselves” (= 0).

## Discussion

With this experiment we aimed to explore how undermining belief in free will affects altruistic behavior in terms of charitable giving. On the basis of previous studies [17,18] that found free will disbelief to be associated with reduced prosocial behavior we expected that undermining people’s belief in free will would reduce the probability that subjects would select the more generous distribution. Our results indicate, however, that this was not the case. While our treatment did reduce belief in free will by 8.9 points on a 100-point scale, this did not significantly influence the likelihood of subjects selecting the more generous distribution. This null result is robust to controlling for sex, age, perceived socio-economic status, task characteristics and the order in which the decision tasks were presented.

Contrary to previous experiments on the effects of free will beliefs, we did not work with a sample of college students, but included a more diverse population. Whereas the average treatment effect was insignificant, our results indicate that the treatment did significantly reduce charitable giving among non-religious subjects. We considered several possible explanations for why religious people seem to be buffered from the treatment effect of our experiment. One possible explanation is that religious people are less open to scientific evidence, and thus less easily influenced by the free will disbelief treatment, which consisted of the presentation of neuroscientific evidence. However, this does not seem plausible as the treatment equally affected the belief in free will among both religious and non-religious subjects. Another explanation we explored is related to religion-based social norms. In line with previous studies that show an association between religious affiliation and the socialization of social norms promoting the helping of others [47–50], our results indicate that religious subjects had a stronger adherence to the social norm of helping the poor. More strongly identifying with this norm might buffer religious people against the effect of undermining belief of free will on charitable giving. Since our treatment was equally effective in reducing belief in free will amongst religious and non-religious people, we conclude that among religious subjects the negative effects of free will disbelief on charitable giving were cancelled out by their adherence to religion-based helping norms. The notion that religion-based helping norms affect giving behavior is further supported by our finding that only non-religious subjects’ choices are influenced by the opportunity cost of the more altruistic option compared to the other option, whereas religious subjects’ decisions are not influenced by this opportunity cost.

### Limitations and strengths

Several limitations apply to our study. Firstly, we have limited insight into the mechanisms by which religious affiliation might moderate the influence of free will beliefs. Although our data point towards the role of helping norms, we cannot exclude the possibility that other aspects of religious affiliation play a role. Future research in which both free will beliefs and the salience of religion-based helping norms are manipulated could shed more light on this. Compared to the main other study that used an experiment to investigate the effect of free will belief on altruistic behavior, by Baumeister and colleagues [18], our study has the advantage that it measures actual behavior rather than self-reported behavior in hypothetical scenarios. As previous research has indicated a significant bias in self-reported donation behavior [51], we think our study design offers a more reliable estimate of the effect of free will beliefs on charitable giving.

### Practical implications and future research

Our results warrant further caution for drawing the simplistic conclusion that a reduction of free will beliefs will automatically undermine pro-social behaviors. For one, our null result suggests that the previously reported finding that undermining free will belief reduces pro-social behaviors reported might be more nuanced. Second, our finding that this effect only occurs among non-religious subjects suggests that beliefs regarding free will do not operate in isolation, but rather interact with pre-existing social norms and religious beliefs. As our sample of religious subjects consisted largely of Christians, it would be interesting to test the effect of free will belief manipulation among other subjects adhering to other religions. Furthermore, as free will disbelief can promote appreciation for the lack of control others have over their situation, another interesting avenue for future research would be to investigate how free will disbelief affects social preferences towards others that vary in perceived “helplessness”, along the lines of an experiment where subjects could donate towards different kinds of welfare recipients that varied in their degree of perceived deservingness of welfare aid [26].

In sum, our study shows that undermining free will beliefs reduces charitable giving in binary dictator games, but that this effect only applies to non-religious subjects. Our results suggest that religion-based adherence to helping norms might interact with belief in free will and jointly shape altruistic behavior. Future research should shed further light on how belief in free will interacts with pre-existing social norms, such as the norm to help the poor.

## Supporting information

S1 Appendix Experimental instructions. (DOCX)
