# Supplementary material for: Free to help? An experiment on free will belief and altruism
Source: PLoS One. 2017 Mar 10;12(3):e0173193. doi: 10.1371/journal.pone.0173193 (PMC5345790; doi:10.1371/journal.pone.0173193)
Supplement: S1 Appendix — (DOCX) [file pone.0173193.s001.docx]

**Supporting information: S1 Appendix**

Subjects were shown the following screens during the experiment which was conducted online through the Amazon mTurk platform

**Page 0 (Welcome and informed consent)**

Principal Investigator: Dr. Jonathan Schooler, Department of Psychology, University of California, Santa Barbara Phone: (805) 893-5969

You have been asked to participate in a 2-part study, supervised by Dr. Jonathan Schooler, a psychology professor at the University of California at Santa Barbara. The first part of the study is about differences in mood following exposure to passages of text. You will then engage in a second study in which you can earn money. We will provide you with specific instructions prior to the starting the study.

The study will take approximately 10 minutes and you will receive $1 in exchange for your participation. Additionally, there is also the opportunity for you to receive up to an additional $1 based on the outcome of the second part of the study (Rewards will be granted through M-Turk's "Bonus" system at the same time the Hit is approved). You will only get an opportunity to receive this money if you have filled out all questions.

Your participation in this research will be kept strictly confidential. To preserve your anonymity, you will be provided with a Participant ID number. Any information that you provide will be available only to members of the research team for approved research purposes. If you feel uncomfortable at any time during the study please inform us immediately. Participation in this study is voluntary and you are free to discontinue your participation at any time.

If you decide not to participate, your refusal will involve no penalty and you will still receive full compensation. If you have any questions about this research or concerns about your participation, please contact the Human Subjects Committee at (805) 893-3807 or hsc@research.ucsb.edu. Or write to the University of California, Human Subjects Committee, Office of Research, Santa Barbara, CA 93106-2050

Participation in research is voluntary. Checking the box below will indicate that you have decided to participate as a research subject in the study described above. You may request an original and dated copy of this form to keep. Checking the box below indicates that you consent to participate in this study.

- By checking this box I agree that I have read and understood the terms and conditions above and certify that I am at least 18 years of age or older

**Page 1 (Happiness question)**

How happy do you feel at this very moment?   Please indicate with the slider below your current level of happiness (0=very unhappy, 10=very happy)

______ Current happiness

**Page 2 (Information treatment, Treatment group)**

We now ask you to read the following passage from the magazine "New Scientist"   Please read it carefully as we will afterwards ask you to write a short summary of the text. If your summary doesn't relate to the text this might affect your payment.

---


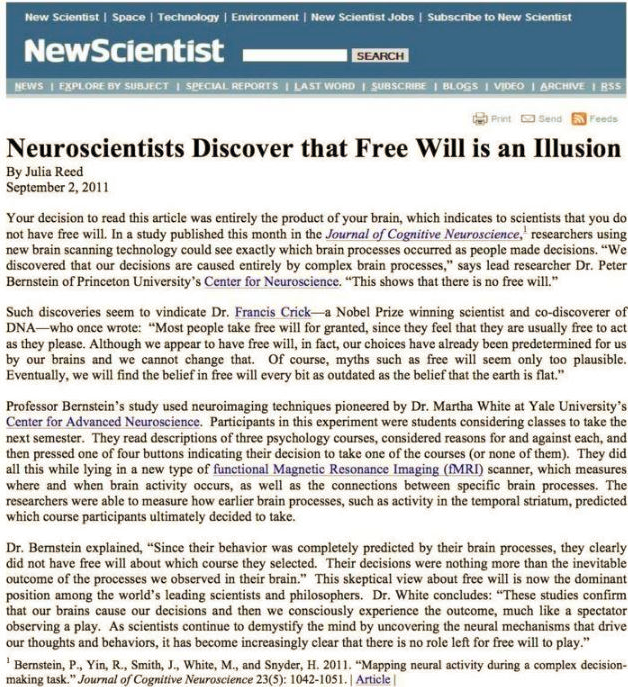


---

Please write a very short summary of the text (1-2 sentences):

**Page 2 (Information treatment, Control group)**

We now ask you to read the following passage from the magazine "New Scientist"   Please read it carefully as we will afterwards ask you to write a short summary of the text. If your summary doesn't relate to the text this might affect your payment.

---


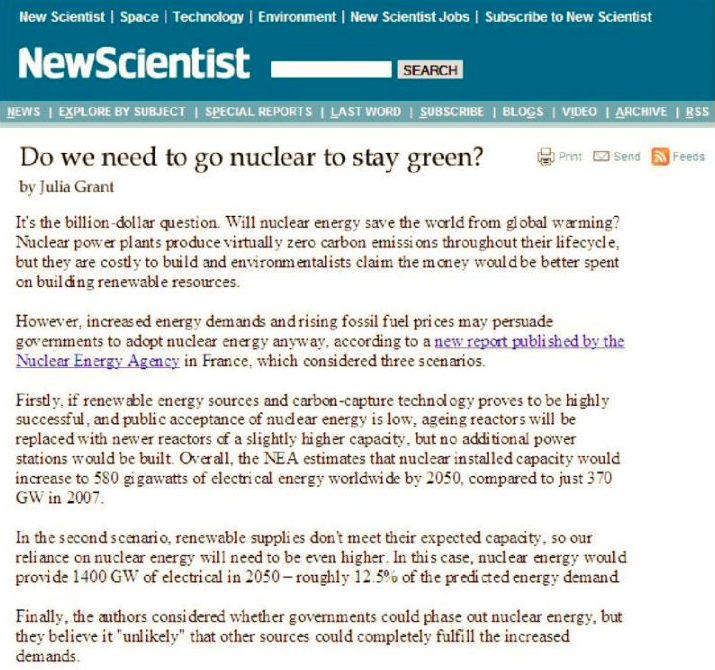


**Page 3 (Happiness question)**

Please consider again how happy you feel at this very moment (0=very unhappy, 10=very happy)

______ Current happiness

**Page 4 (Instructions to decision task)**

We will now proceed to a part of the study where you can earn additional money. You can earn up to 100 tokens, each token being worth 1 dollar cent.   There are 24 decision-tasks. In each task you are asked to select 1 out of 2 possible allocations. By selecting an allocation, you decide how tokens are divided between you and another person.    This other person is a poor person in a developing country, who will be selected by the charity “GiveDirectly”. This charity directly sends cash to impoverished people in developing countries. An example of a person that is supported by GiveDirectly is Beatrice (31years) from Kenya, who has two young children and no income.

*[Here, subjects are shown a picture of Beatrice and her children]*

At the end of the study, 1 decision-task from the list of 24 decision-tasks will be randomly selected to be paid out in real money. Based on which allocation you selected in this choice, both you and/or the poor person will receive some amount of money. It is thus important that you pay close attention in all 24 choice-tasks! You have 10 seconds for each decision-task. If you fail to make a choice within this time neither you nor the poor person can receive any money in case this choice is selected.

**Page 4 (Test questions)**

Before we begin with the decision-task please answer a few questions to confirm that you have understood the instructions. Your answers to these questions do not influence your payment.

Can you earn real money with the decision-tasks?

- Yyes
- No

You will allocate money between yourself and another person. Who is this other person?

- A poor person in a developing country
- Another m-Turk participant in this experiment

**Page 5 (Decision tasks)**

*[Example screenshot below, full series of decision-tasks shown in table1*]


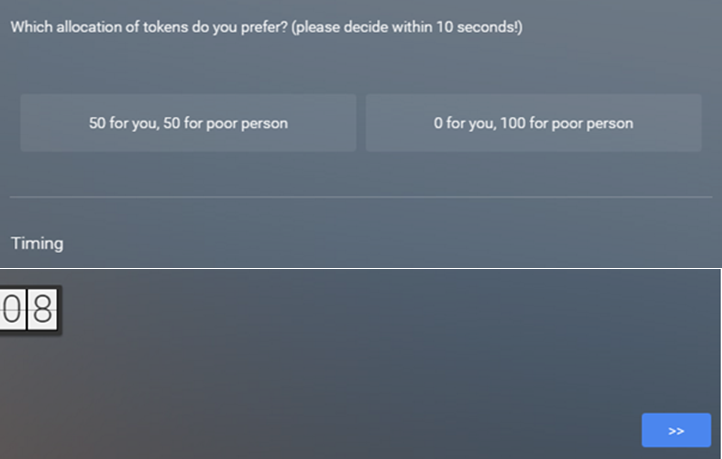


**Page 6 (Attention check)** To check you're still paying attention, please answer the following question: What is 2+3?

______

**Page 7 (Demographic survey)**

What is your age?

- 18-29
- 30-39
- 40-49
- 50-59
- 60 and above

Are you a male or female?

- Male
- Female

Do you consider yourself as belonging to any particular religion or denomination?

- No
- Yes, Roman Catholic
- Yes, Protestant
- Yes, other Christian denomination
- Yes, Jewish
- Yes, Muslim
- Yes, Hindu
- Yes, Buddhist
- Yes, other, please specify:

Please consider the following ladder. The bottom represents the least successful people in society, the top represents the most successful people. Where do you see yourself on this ladder (0=least successful, 10=most successful)?

Position on ladder (0-10)

**Page 8 (Manipulation check)**

Please consider the following statement:      "I fully believe I have free will"     Please indicate below to what extent you agree with this statement:

______ 0=totally disagree, 100=totally agree

**Page 9 (Control questions)**

When deciding how much to donate to GiveDirectly, did you consider whether Beatrice has any control over her lack of income?

- Yes
- No

Thinking about it now, how much control do you think Beatrice has over her lack of income?

______ 0=no control, 100=complete control

Which of the following two statements do you most agree with?

- One ought to help people such as Beatrice

People such as Beatrice ought to help themselves
